# Lived experiences with online group psychosocial support among dementia caregivers and service providers

**DOI:** 10.1002/alz70858_097773

**Published:** 2025-12-24

**Authors:** Yuhan Huang, Jinsong Lang, Huali Wang

**Affiliations:** ^1^ Communication University of China, Beijing, Beijing, China; ^2^ NHC Key Laboratory for Mental Disorders, Beijing, Beijing, China; ^3^ Dementia Care and Research Center, Peking University Institute of Mental Health (Sixth Hospital), Beijing, China; ^4^ National Clinical Research Center for Mental Disorders, Beijing, China

## Abstract

**Background:**

With the development of digital technology, people increasingly use online communication platforms. More and more family dementia caregivers are beginning to receive online psychosocial support. The lived experiences in online psychosocial support activities may influence the preference for seeking support online or offline among dementia caregivers. Therefore, the presented study aimed to explore the subjective experiences of social support among family dementia caregivers and service providers on online psychosocial support.

**Method:**

Sixteen family dementia caregivers and four service providers participated in the in‐depth qualitative interviews. The semi‐structured interview guideline inquired about their experiences participating in the online caregiver support group, including their feeling of acquiring knowledge and support, sources of support, and substantial and emotional support. In addition to the thematic analysis of lived experiences of online psychosocial support, the difference between attending offline and online group support was investigated. Researchers used participant observation to analyze their performance and behavior as online group members.

**Results:**

Online psychosocial support for caregivers is relatively simple and easy to access, but the prerequisite for obtaining such support is whether to receive peer support through online interactions, establish trust relationships and emotional connections, and expand social networks. Therefore, gaining psychosocial support is a process that also requires time and the ability to use social media. However, caregivers felt that participation in online psychosocial support somewhat weakened their interactivity. Expression of their emotional experiences and participation in interactions and communications were also constrained by online technologies, sometimes resulting in the breakdown of social support networks. In contrast, offline memory cafés allow more caregivers to express their emotions and pressures fully. They can obtain the help they desire and even share their experiences with other families without time and space constraints.

**Conclusion:**

The study finds that, though taking advantage of accessibility, online psychological support faces challenges in fulfilling emotional support and facilitating interaction among family caregivers. The findings facilitate our understanding of the theory of social support networks in the context of social media.

